# Dimethyl Fumarate Used as an Effective Treatment for Granuloma Annulare Disseminatum: An Immunohistochemical Case Study

**DOI:** 10.3390/ijms241713355

**Published:** 2023-08-28

**Authors:** Max Gabutti, Kristine Heidemeyer, S. Morteza Seyed Jafari, Simon Bossart, Robert E. Hunger, Laurence Feldmeyer, Nikhil Yawalkar

**Affiliations:** Department of Dermatology, Inselspital, Bern University Hospital, University of Bern, 3010 Bern, Switzerland; max.gabutti@insel.ch (M.G.); morteza.jafari@insel.ch (S.M.S.J.); simon.bossart@insel.ch (S.B.); robert.hunger@insel.ch (R.E.H.); laurence.feldmeyer@insel.ch (L.F.); nikhil.yawalkar@insel.ch (N.Y.)

**Keywords:** granuloma annulare disseminatum, inflammatory cells, dimethyl fumarate

## Abstract

This investigation demonstrates the use of dimethyl fumarate (DMF) for the treatment of disseminated granuloma annulare (GAD), a rare and chronic inflammatory skin disease. In this case, progressive GAD was treated with DMF, resulting in significant improvement of skin lesions within 5 weeks and complete healing within 7 months. Clinical response was associated with a reduction in inflammatory cells, including both T cell subsets (CD4+ > CD8+), CD183^+^/CXCR3^+^ cells, Langerhans cells (CD1a+), myeloid DCs, M1- and M2-like macrophages and the activation marker HLA-DR in immunohistochemical analysis. These findings support the use of DMF as a promising treatment option for this rare skin condition.

## 1. Introduction

Granuloma annulare (GA) is a benign inflammatory skin disease. Localized GA is likely to resolve spontaneously, while generalized GA is rare and may persist for decades [1]. Disseminated GA is characterized by widespread erythematous papules and is often chronic and difficult to treat [1,2]. In disseminated GA, systemic treatment may be required [2]. The successful treatment of disseminated GA when using topical tacrolimus and pimecrolimus, psoralen plus ultraviolet A (PUVA) and systemic agents like cyclosporine, dapsone, hydroxychloroquine, isotretinoin, niacinamide, potassium iodide, vitamin E or TNF-alpha blockers has been reported [1,2,3]. None of these therapies listed is effective in more than 50% of patients, and some may have severe side effects. Therefore, there is a need for an optimized therapy with little or no side effects [2]. In some reports, fumaric acid esters have been shown to be an effective treatment option in the management of disseminated GA [2,4,5]. In the current study, we showed a good clinical response to dimethyl fumarate (DMF, Skilarence^®^) for granuloma annulare disseminatum with a focus on the immune mechanisms underlying treatment efficacy.

## 2. Case Report and Results

**Figure 1:**
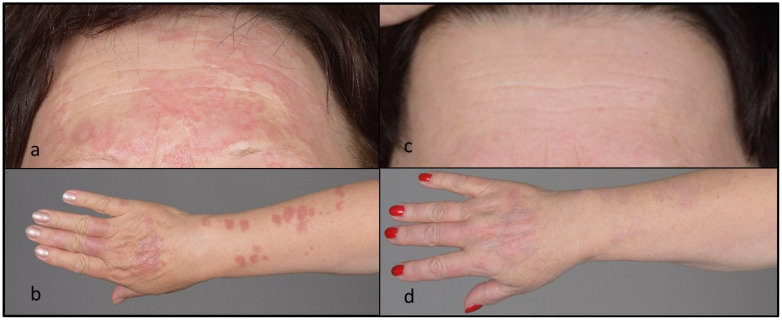
Clinical image; (**a**,**b**): widespread, erythematous papules and plaques on the arms and face; (**c**,**d**): after treatment with DMF.

**Figure 2:**
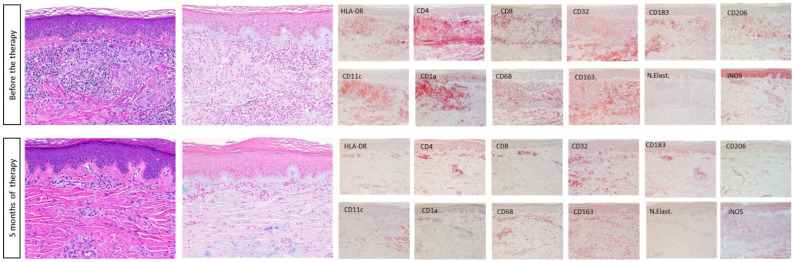
Histopathological analysis of the skin lesions with additional immunohistochemical stainings before and 5 months after start of therapy with DMF. Original magnification H&E × 200; IHC × 200.

A 61-year-old female was referred to our hospital for progressive skin lesions since about 1 year previously. The lesions were mostly asymptomatic but had gradually increased in size and extent. Upon examination, there were numerous erythematous plaques involving the face and upper extremities (Figure 1). Histological (haematoxylin and eosin (H&E), colloidal iron) stainings of a lesional punch biopsy specimen were performed. Histopathological analysis of the skin lesions revealed perivascular, partly interstitial inflammation with numerous giant cells and small and focal mucin-rich necrobiosis areas (Figure 2). Histopathological examination confirmed the diagnosis of granuloma annulare and excluded further granulomatous diseases such as sarcoidosis, infections or drug-induced granulomatous dermatosis. Previous therapies, including topical corticosteroids, topical calcineurin inhibitors (tacrolimus ointment), and methotrexate (7.5–17.5 mg s.c./week for about 2 months, which was stopped due to the aggravation of skin lesions and elevated liver enzymes), were used unsuccessfully. Therefore, DMF (progressive therapy scheme to 240 mg–120 mg–120 mg/day) was started, which resulted in improvement of the cutaneous lesions within 5 weeks and complete healing within 7 months. The doses were then gradually reduced to 120 mg/day, also due to a light lymphocytopenia (1.2 × 10^3^/mL; normal range: 1.4–4.8 × 10^3^/mL). No further side effects were observed. After around one year, DMF could be slowly tapered without recurrence of skin lesions over 3 years of follow-up.

To better understand the mode of action of DMF, immunohistopathological stainings were performed and evaluated. As shown in Figure 2, the inflammatory infiltrate in the skin lesion prior to treatment with DMF consisted of a high number of T cells (CD4+ > CD8+) and CD183^+^/CXCR3^+^ cells (representing a marker for type 1 T cells). An enhanced number of dendritic cell (DC) and macrophage subsets were also variably distributed within the skin lesion. Whereas Langerhans cells (CD1a^+^) were mainly seen in the epidermis and upper dermis, myeloid DCs (CD11c^+^), M1-like (CD68^+^, CD32^+^, HLA-DR^+^, iNOS^+^) and M2-like macrophages (CD163^+^, CD206^+^) were observed in the dermal cell infiltrate. In contrast, only a few neutrophils were detected throughout the cell infiltrate. The histological and immunohistochemical assessment showed a substantial reduction in inflammatory cells 5 months after the initiation of the therapy with DMF, which could explain the clinical efficacy of the therapy. In particular, a marked reduction in both T cell subsets (CD4+ > CD8+), CD183^+^/CXCR3^+^ cells, Langerhans cells (CD1a+), myeloid DCs (CD11c^+^) and both M1- and M2-like macrophages, as well as the activation marker HLA-DR, was observed.

## 3. Discussion

GA has been suggested to represent a delayed-type hypersensitivity (type 1 T cells) reaction contributing to the activation of macrophages expressing the tumour necrosis factor alpha and matrix metalloproteinase reaction, resulting in matrix degradation [6]. As shown here, GA is characterized by lymphohistiocytic and monocytic infiltrates that form palisading granulomas with central necrobiotic changes. Numerous apoptotic macrophages have been observed within the necrobiotic areas [6]. Disseminated GA is a rare disease, and no reproducible effective treatment has been established, with the exception of topical glucocorticoids for localized applications [2]. The described systemic therapies are all immunosuppressive with potential side effects [2]. Biologics, including TNFα-inhibitors, have shown good efficacy and safety in various inflammatory skin diseases [7]. Adalimumab has shown clinical response in up to 80% of GA patients [3]. However, high costs, the risk of adverse events, such as infections, and the need for injections are limiting factors. DMF is a treatment for moderate-to-severe psoriasis and multiple sclerosis. DMF therapy typically improves skin inflammation within the first 3 months of treatment [8].

Similarly to our study, fumaric acid esters (FAEs) have been proposed as an alternative treatment option in GGA in previous reports [1,4,6]. In our patient, the therapy could be optimized and tapered without relapse of disease, as has already been reported for other systemic therapies [9]. FAEs appear to shift a T-helper-cell 1-directed immune response towards a T-helper-cell 2 type of immune response [1,4,6,10]. In addition, fumarates modulate T cell activation by reducing interleukin 12 and type 1 cytokines like interferon gamma, with simultaneous pronounced stimulation of the Th2 cytokines, such as interleukin 4, 5 or 10 [2,6,10]. In accordance with this, we found a marked reduction in CD183/CXCR3, which is particularly expressed in type 1 T cells. Furthermore, the antipsoriatic activity of FAE may also be mediated by diminishing proinflammatory cytokine overexpression and the antigen-presenting capacity of monocytes and macrophages. FAE induces apoptosis in human monocyte-derived dendritic cells as well as keratinocytes [6,10].

## 4. Methods

To study the mechanism of action of DMF in GA, immunohistochemical stainings were performed in pre- and post-treatment (at 5 months) skin biopsy specimens using the avidin–biotin complex–alkaline phosphatase (ABC-AP) method. The following primary antibodies were used: CD1a (clone MTB1; Leica Biosystems, Nussloch, Germany), CD4 (clone 4B12; DakoCytomation, Glostrup, Denmark), CD8 (clone 4B11; Leica Biosystems), CD11c (clone 5D11; Novocastra, Muttenz, Switzerland), CD32 (clone EPR6657; Abcam, Cambridge, MA, USA), CD68 (clone PG-M1, DakoCytomation), CD163 (clone EDHU-1; Serotec MCA, Oxford, UK), CD 183 (clone 1C6/C-X-C motif chemokine receptor 3 (CXCR3); BD Pharmingen, San Diego, CA, USA), CD 206 (HPA045134, Sigma, Heidelberg, Germany), inducible nitric oxide synthase (iNOS; clone EPR16635, 80 Abcam, Cambridge, UK), neutrophil elastase (clone NP57; DakoCytomation) and HLA-DR (clone TAL.1B5; DakoCytomation). Irrelevant immunoglobulin G subclass-matched antibodies were used for negative controls.

## 5. Conclusions

Fumaric acid esters, especially DMF, as a relatively new medication in this group, could be a promising alternative for the management of recalcitrant disseminated GA. However, large controlled trials are needed to analyse the efficacy and safety of treatment with FAEs in patients with DGA.

## Data Availability

The datasets presented in this article are not readily available due to ethical/privacy restrictions. Requests to access the datasets should be directed to the corresponding author.
